# Machine-learning phenotyping of patients with functional mitral regurgitation undergoing transcatheter edge-to-edge repair: the MITRA-AI study

**DOI:** 10.1093/ehjdh/ztaf006

**Published:** 2025-02-13

**Authors:** Fabrizio D’Ascenzo, Filippo Angelini, Corrado Pancotti, Pier Paolo Bocchino, Cristina Giannini, Filippo Finizio, Marianna Adamo, Victoria Camman, Nuccia Morici, Leor Perl, Saverio Muscoli, Gabriele Crimi, Paolo Boretto, Ovidio de Filippo, Luca Baldetti, Giuseppe Biondi-Zoccai, Federico Conrotto, Sonia Petronio, Arturo Giordano, Rodrigo Estévez-Loureiro, Davide Stolfo, Christian Templin, Mauro Chiarito, Elena Cavallone, Veronica Dusi, Gianluca Alunni, Jacopo Oreglia, Mario Iannaccone, Marco Pocar, Matteo Pagnesi, Stefano Pidello, Ran Kornowski, Piero Fariselli, Simone Frea, Michele La Torre, Claudia Raineri, Giuseppe Patti, Italo Porto, Antonio Montefusco, Sergio Raposeiras Roubin, Gaetano Maria De Ferrari

**Affiliations:** Division of Cardiology, Cardiovascular and Thoracic Department, ‘Citta della Salute e della Scienza’ Hospital, Corso Bramante n 88, Turin 10126, Italy; Department of Medical Sciences, University of Turin, Via Verdi 8, Turin 10126, Italy; Division of Cardiology, Cardiovascular and Thoracic Department, ‘Citta della Salute e della Scienza’ Hospital, Corso Bramante n 88, Turin 10126, Italy; Department of Medical Sciences, University of Turin, Via Verdi 8, Turin 10126, Italy; Department of Medical Science, University of Turin, Via Santena 19, 10126 Torino, Italy; Division of Cardiology, Cardiovascular and Thoracic Department, ‘Citta della Salute e della Scienza’ Hospital, Corso Bramante n 88, Turin 10126, Italy; Department of Medical Sciences, University of Turin, Via Verdi 8, Turin 10126, Italy; IRCCS Fondazione Don Carlo Gnocchi ONLUS, Milan 20148, Italy; Division of Cardiology, Mediterranea Cardiocentro, Napoli, Italy; Faculty of Medical and Health Sciences, Tel Aviv University, Tel Aviv, Israel; Department of Cardiology, University Heart Center, University Hospital Zurich, Zurich, Switzerland; Division of Cardiology, San Giovanni Bosco Hospital, ASL Città di Torino, Torino, Italy; Cardiac Intensive Care Unit and De Gasperis Cardio Center, ASST Grande Ospedale Metropolitano Niguarda, Milan, Italy; Faculty of Medical and Health Sciences, Tel Aviv University, Tel Aviv, Israel; Cardiology Department, Rabin Medical Center, Petah Tikva, Israel; Cardiovascular Interventional Operative Unit, Presidio Ospedaliero Pineta Grande, Castel Volturno, Caserta, Italy; Operative Unit of Hemodynamics, Casa di Salute Santa Lucia, San Giuseppe Vesuviano, Naples, Italy; Division of Cardiology, Cardiovascular and Thoracic Department, ‘Citta della Salute e della Scienza’ Hospital, Corso Bramante n 88, Turin 10126, Italy; Department of Medical Sciences, University of Turin, Via Verdi 8, Turin 10126, Italy; Division of Cardiology, Cardiovascular and Thoracic Department, ‘Citta della Salute e della Scienza’ Hospital, Corso Bramante n 88, Turin 10126, Italy; Department of Medical Sciences, University of Turin, Via Verdi 8, Turin 10126, Italy; Department of Biomedical Sciences, Humanitas University, Pieve Emanuele, Milan, Italy; Department of Medical-Surgical Sciences and Biotechnologies, Sapienza University of Rome, Latina, Italy; Maria Cecilia Hospital, GVM Care and Research, Cotignola, Italy; Division of Cardiology, Cardiovascular and Thoracic Department, ‘Citta della Salute e della Scienza’ Hospital, Corso Bramante n 88, Turin 10126, Italy; Department of Medical Sciences, University of Turin, Via Verdi 8, Turin 10126, Italy; Division of Cardiology, Cardiovascular and Thoracic Department, ‘Citta della Salute e della Scienza’ Hospital, Corso Bramante n 88, Turin 10126, Italy; Department of Medical Sciences, University of Turin, Via Verdi 8, Turin 10126, Italy; Cardiovascular Interventional Operative Unit, Presidio Ospedaliero Pineta Grande, Castel Volturno, Caserta, Italy; Cardiology Department, University Hospital Álvaro Cunqueiro, Estrada de Clara Campoamor, 341, Vigo, Pontevedra 36213, Spain; Department of Anaesthesia and Intensive Care, IRCCS San Raffaele Scientific Institute, Milan, Italy; Cardiothoracovascular Department, Azienda Sanitaria Universitaria Giuliano Isontina (ASUGI) and University Hospital of Trieste, Trieste, Italy; Faculty of Medical and Health Sciences, Tel Aviv University, Tel Aviv, Israel; Division of Cardiology, Department of Systems Medicine, Tor Vergata University, Rome 00133, Italy; Cardiology and Cardiac Catheterization Laboratory, Department of Medical and Surgical Specialties, Radiological Sciences, and Public Health, Civil Hospitals, University of Brescia, Brescia, Italy; Division of Cardiology, Cardiovascular and Thoracic Department, ‘Citta della Salute e della Scienza’ Hospital, Corso Bramante n 88, Turin 10126, Italy; Department of Medical Sciences, University of Turin, Via Verdi 8, Turin 10126, Italy; Division of Cardiology, Cardiovascular and Thoracic Department, ‘Citta della Salute e della Scienza’ Hospital, Corso Bramante n 88, Turin 10126, Italy; Department of Medical Sciences, University of Turin, Via Verdi 8, Turin 10126, Italy; Division of Cardiology, Cardiovascular and Thoracic Department, ‘Citta della Salute e della Scienza’ Hospital, Corso Bramante n 88, Turin 10126, Italy; Department of Medical Sciences, University of Turin, Via Verdi 8, Turin 10126, Italy; Dipartimento Universitario di Medicina Traslazionale, Università Piemonte Orientale, Azienda Ospedaliero-Universitaria Maggiore della Carità di Novara, Novara, Italy; Department of Internal Medicine (DIMI), University of Genoa, Genova, Italy; Department of Cardiology, University Heart Center, University Hospital Zurich, Zurich, Switzerland; Cardiac Catheterization Laboratory, Cardiothoracic and Vascular Department, Azienda Ospedaliero Universitaria Pisana, Pisa, Italy; Division of Cardiac Surgery, Department of Surgical Sciences, University of Turin, Turin, Italy; Division of Cardiology, Cardiovascular and Thoracic Department, ‘Citta della Salute e della Scienza’ Hospital, Corso Bramante n 88, Turin 10126, Italy; Department of Medical Sciences, University of Turin, Via Verdi 8, Turin 10126, Italy; IRCCS Fondazione Don Carlo Gnocchi ONLUS, Milan 20148, Italy; Division of Cardiology, San Giovanni Bosco Hospital, ASL Città di Torino, Torino, Italy; Department of Medical Science, University of Turin, Via Santena 19, 10126 Torino, Italy; Division of Cardiology, Cardiovascular and Thoracic Department, ‘Citta della Salute e della Scienza’ Hospital, Corso Bramante n 88, Turin 10126, Italy; Department of Medical Sciences, University of Turin, Via Verdi 8, Turin 10126, Italy; Division of Cardiac Surgery, Department of Surgical Sciences, University of Turin, Turin, Italy; Division of Cardiology, Cardiovascular and Thoracic Department, ‘Citta della Salute e della Scienza’ Hospital, Corso Bramante n 88, Turin 10126, Italy; Department of Medical Sciences, University of Turin, Via Verdi 8, Turin 10126, Italy; Cardiovascular Interventional Operative Unit, Presidio Ospedaliero Pineta Grande, Castel Volturno, Caserta, Italy; Cardiovascular Interventional Operative Unit, Presidio Ospedaliero Pineta Grande, Castel Volturno, Caserta, Italy; Division of Cardiology, Cardiovascular and Thoracic Department, ‘Citta della Salute e della Scienza’ Hospital, Corso Bramante n 88, Turin 10126, Italy; Department of Medical Sciences, University of Turin, Via Verdi 8, Turin 10126, Italy; Maria Cecilia Hospital, GVM Care and Research, Cotignola, Italy; Division of Cardiology, Cardiovascular and Thoracic Department, ‘Citta della Salute e della Scienza’ Hospital, Corso Bramante n 88, Turin 10126, Italy; Department of Medical Sciences, University of Turin, Via Verdi 8, Turin 10126, Italy

**Keywords:** Machine-learning, Artificial intelligence, Mitral regurgitation, Transcatheter mitral valve repair, MitraClip

## Abstract

**Aims:**

Severe functional mitral regurgitation (FMR) may benefit from mitral transcatheter edge-to-edge repair (TEER), but selection of patients remains to be optimized.

**Objectives:**

The aim of this study was to use machine-learning (ML) approaches to uncover concealed connections between clinical, echocardiographic, and haemodynamic data associated with patients’ outcomes.

**Methods and results:**

Consecutive patients undergoing TEER from 2009 to 2020 were included in the MITRA-AI registry. The primary endpoint was a composite of cardiovascular death or heart failure (HF) hospitalization at 1 year. External validation was performed on the Mitrascore cohort. 822 patients were included. The composite primary endpoint occurred in 250 (30%) patients. Four clusters with decreasing risk of the primary endpoint were identified (42, 37, 25, and 20% from Cluster 1 to Cluster 4, respectively). Clusters were combined into a high-risk (Clusters 1 and 2) and a low-risk phenotype (Clusters 3 and 4). High-risk phenotype patients had larger left ventriculars (LVs) (>107 mL/m^2^), lower left ventricular ejection fraction (<35%), and more prevalent ischaemic aetiology compared with low-risk phenotype patients. Within low-risk groups, permanent atrial fibrillation amplified that of HF hospitalizations. In the Mitrascore cohort, the incidence of the primary endpoint was 48, 52, 35, and 42% across clusters.

**Conclusion:**

A ML analysis identified meaningful clinical phenotypic presentations in FMR undergoing TEER, with significant differences in terms of cardiovascular death and HF hospitalizations, confirmed in an external validation cohort.

## Graphical Abstract

**Graphical Abstract:**
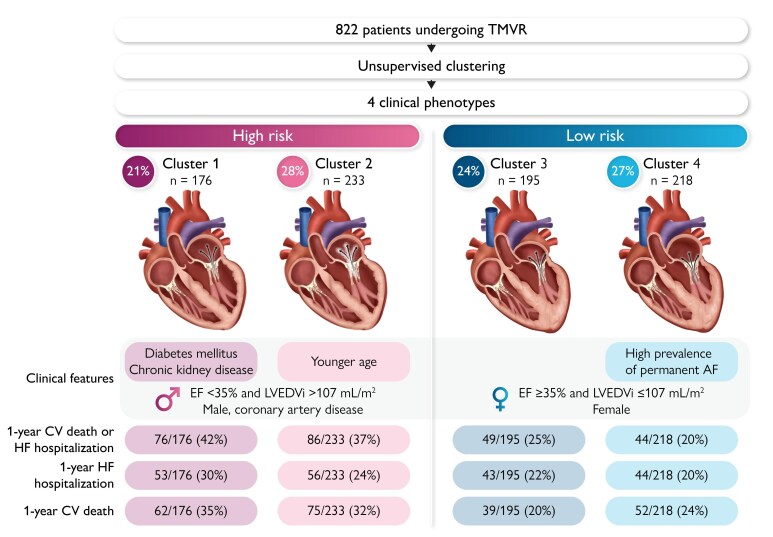

## Introduction

Mitral regurgitation (MR) is a common disease, affecting up to 22% of elderly patients worldwide.^1^ Severe functional MR (FMR), developing in the absence of clear valvular structural abnormalities, typically occurs in the context of left-sided chambers dilation and dysfunction and, when ‘disproportionate’ to the left ventricular (LV) end-diastolic volume, has been shown to benefit from transcatheter edge-to-edge repair (TEER) with the MitraClip device.^2^ However, this proportionality framework has been variably questioned in both its short-term and long-term reliability.^3^ Moreover, this concept does not consider clinical data, atrial contribution to FMR, and haemodynamic parameters, which may all help identifying durable responders to TEER highlighting the need of a careful clinical judgement with a multi-parametric approach.^4–6^

Machine-learning (ML) approaches can uncover concealed connections between clinical, echocardiographic, and haemodynamic data and refine our knowledge of complex cardiovascular conditions and their response to therapy^7–9^ and a recent artificial intelligence-derived risk score has been developed to improve prognostication.^10^ Aims of the present study were to use ML to identify different informative groups of patients undergoing TEER for at least moderate-to-severe FMR, to explore the clinical, echocardiographic, and haemodynamic characteristics of the different groups and to assess their outcomes.

## Methods

### Data collection

This study is based on the multicentric international Mitra-AI (MitraClip Artificial Intelligence) registry, which included 824 patients referred for TEER for FMR between 2009 and 2020 from nine centres in Europe and Israel (see Supplementary material online, *Appendix*). Adult (≥18 years) patients were eligible for study inclusion. Eligible patients had moderate-to-severe or severe functional MR and had a New York Heart Association (NYHA) functional Classes II–IV despite the use of optimal medical and device therapy for heart failure (HF). Only patients who were alive at hospital discharge were included in the present registry. Successful TEER was defined according to Mitral Valve Academic Research Consortium definition. A complete list of inclusion and exclusion criteria is reported in the Supplementary material online, *Appendix*.

Demographic, clinical, echocardiographic, and haemodynamic data were collected through standardized forms and revision of clinical charts.

The validation of clustering-based model was conducted on the Mitrascore dataset^11^ and on a cohort of patients with moderate-to-severe functional MR treated with medical therapy.^12^

Also, the Mitrascore was calculated on both the derivation cohort and the Mitrascore validation cohort.

The study was conducted according to the Declaration of Helsinki and all patients provided written informed consent for inclusion in the present study. The study protocol was reviewed by the respective local Ethics Committee or Investigational Review Board at each collaboration site.

### Study endpoints

The primary endpoint was a composite of cardiovascular death or HF hospitalizations at 1 year. Secondary endpoints included individual components of the composite primary endpoint. To avoid intrinsic reporting biases of death certificates,^13^ cardiovascular death was adjudicated by medical records revision, when available, and/or telephonic follow-up of close relatives.

### Data pre-processing

The dataset were first standardized by aligning measurement units across centres and by removing variables and records with more than 35% of missing entries. Missing values were imputed using the median for continuous variables and the mode for categorical ones. Subsequently, all variables were normalized. Finally, 11 clinically relevant and easy to measure variables were carefully selected by experts to be included in the clustering-based model: these features were defined ‘a priori^14^’. Variables not included in the model were defined ‘a posteriori.’ The choice to incorporate only a subset of the available variables into the model was driven by the aim to develop a user-friendly tool.

The missing values distribution for the included variables is shown in Supplementary material online, *Figure S1*.

After clustering, the right heart catheterization data were analysed and classified throughout clusters.

### Optimal number of clusters identification

To identify the optimal number of clusters, a K-Medoids with the partition around medoids (PAM) algorithm was performed, varying the number of clusters from 2 to 10.^14^ In this case, the choice of using K-Medoids rather than K-Means is justified by the presence of both continuous and categorical data. For each of the candidate number of clusters the within cluster sum of squared distance (WCSS) and a composite metric based on Silhouette score were calculated.^15^ To obtain robust and significant clusters, we maximized the product of the average silhouette score and the minimum intra-cluster silhouette score. This choice was justified by the fact that the reliance on the average silhouette score alone does not consider the possible imbalance within the intra-cluster silhouette score. In general, the silhouette score is a measure of how each data point is well clustered into a defined group. It is calculated through the mean intra-cluster distance (*a*) and the mean nearest-cluster distance (*b*) for each data point. The distance used in this case was the cosine distance. The silhouette score for a sample is given by (*b* − *a*)/max (*a*, *b*). The number of clusters was chosen based on the maximum of the composite metric together with the elbow method.^16^ The clusters were assigned to the two external validation cohorts to assess the consistency of the observed results. Finally, additional analysis was performed to test the prediction of clustering before and after 2015.

### Baseline statistical analysis

Continuous data are shown as mean ± standard deviation, skewed variables are presented as median (inter-quartile range), and categorical variables are given as numbers and percentages. Comparisons of patients’ baseline characteristics were performed with one-way analysis of variance test for continuous data and the Pearson χ^2^ test for categorical variables. Net reclassification index (NRI) was computed on both derivation and validation cohorts by comparing patients experiencing and not experiencing the primary endpoint stratified by cluster and by classes of Mitrascore (low: score from 0 to 2, intermediate: score from 3 to 4, high: score from 5 to 8).^17^

A two-sided *P*-value < 0.05 was considered statistically significant. All analyses were performed with SPSS version 24.0 (IBM Corporation, Armonk, NY, USA).

## Results

### Study population

From January 2009 through January 2021 a total of 824 patients with moderate-to-severe and severe FMR underwent MitraClip implantation at nine European Centres. The final study population consisted of 822 individuals. Median age was 75 (67–81) years and 298 (36%) patients were women. The median left ventricular ejection fraction (LVEF) was 35% (26–45%). Follow-up was censored at 1 year. The composite primary endpoint occurred in 250 (30%) patients (Supplementary material online, *Table S1*), who were younger [72 (68–75) years vs. 73 (68–76) years, *P* = 0.025] and had a higher prevalence of chronic kidney disease (69 vs. 51%, *P* = 0.005), NYHA Class IV (20 vs. 12%, *P* < 0.001), and diuretics use (98 vs. 91%, *P* < 0.001) compared with the remainder of the study population. As for echocardiographic data, patients facing the composite primary endpoint showed lower LVEF [33% (31–34) vs. 38% (37–390), *P* < 0.001], higher LV end-diastolic volume index [130 (122–138) mL/m^2^ vs. 107 (103–111) mL/m^2^, *P* < 0.001] and more frequent Grade 3 diastolic dysfunction (68 vs. 37%, *P* < 0.001) (Supplementary material online, *Table S2* and Supplementary material online, *Figure S2*). No significant difference was found regarding data from right heart catheterization, which were available for 252 patients overall (Supplementary material online, *Table S3*). Patients facing the composite primary endpoint more frequently had residual moderate MR after MitraClip implantation (10 vs. 4%, *P* < 0.001). The included variables distribution stratified based on the patients’ status (alive vs. dead) are reported in Supplementary material online, *Figure S2*.

### Patients’ clustering

Based on the maximum of the composite metric, in accordance with the elbow of the WCSS, the optimal number of computable phenotypes was 4 (see Supplementary material online, *Figure S3*), with a mean Silhouette coefficient score of 0.49 and an intra-cluster silhouette score of 0.49, 0.46, 0.47, and 0.56, respectively. Both metrics reveal a clear separation among the formed groups.

**Figure 1:**
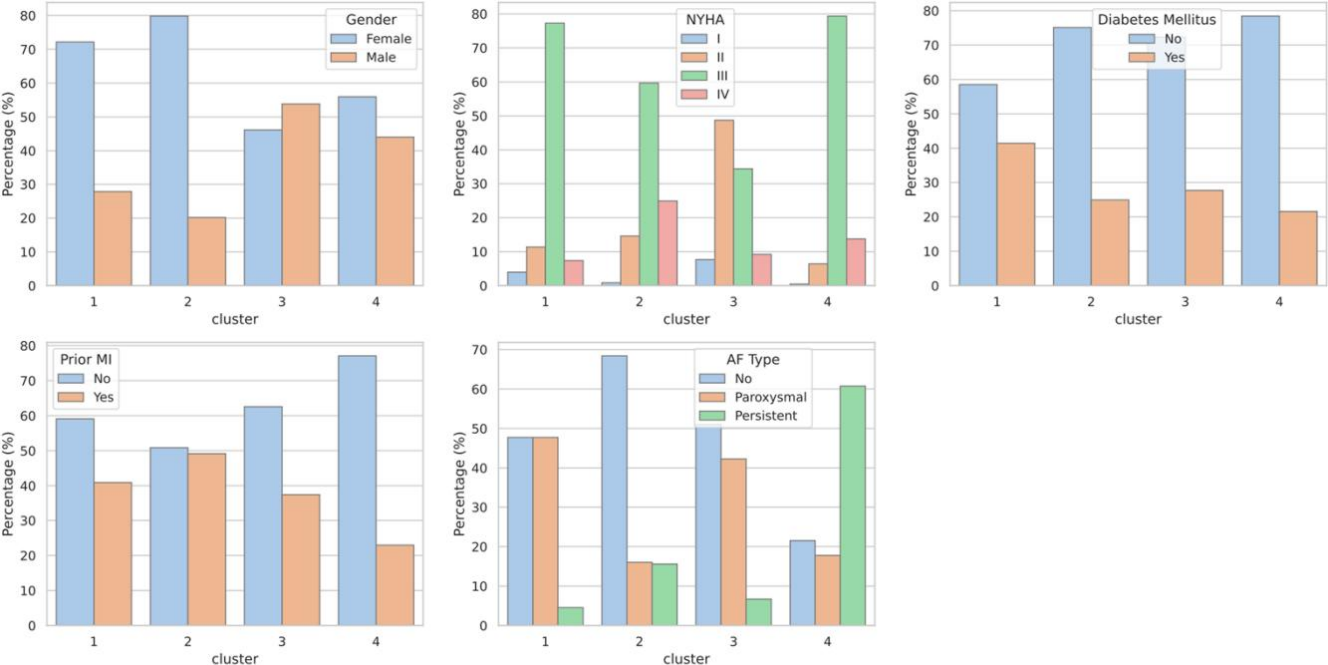
Distribution of dichotomic *a priori* variables according to clusters. AF, atrial fibrillation; MI, myocardial infarction.

**Figure 2:**
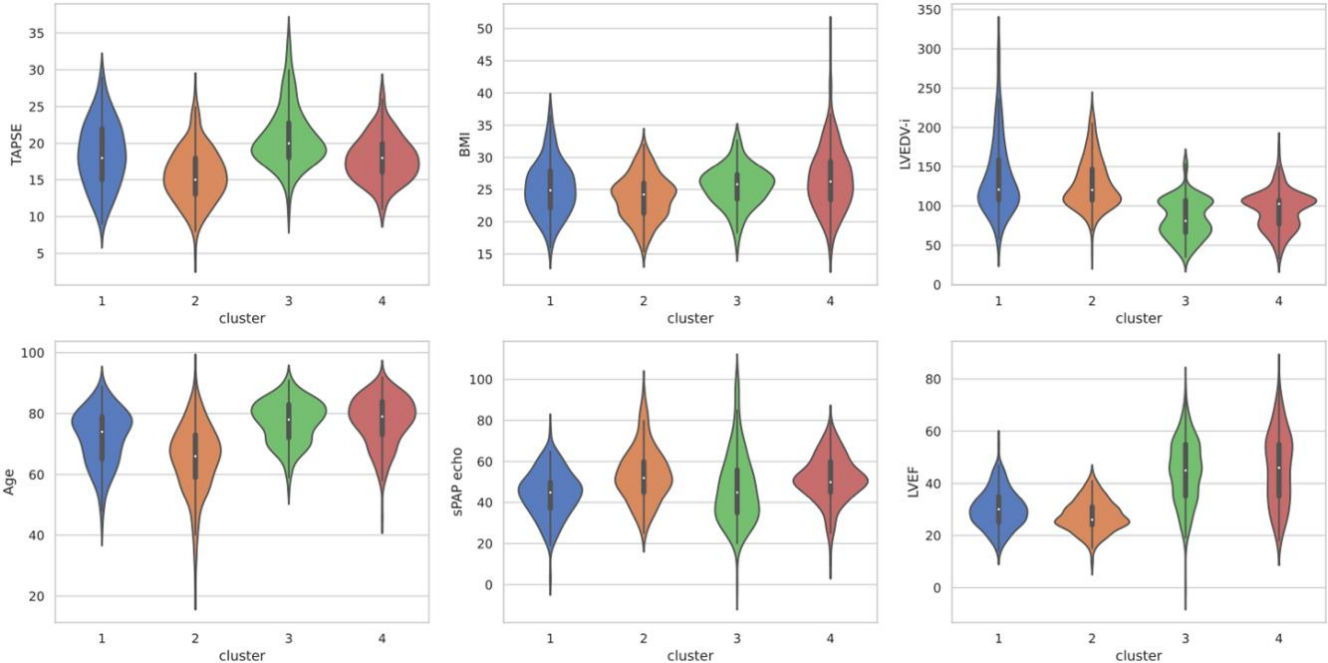
Distribution of continuous variables according to clusters (violin plot). BMI, body mass index; LVEDVi, left ventricle end-diastolic volume index; LVEF, left ventricle ejection fraction; sPAP, systolic pulmonary artery pressure; TAPSE, tricuspid annular plane excursion.

**Figure 3:**
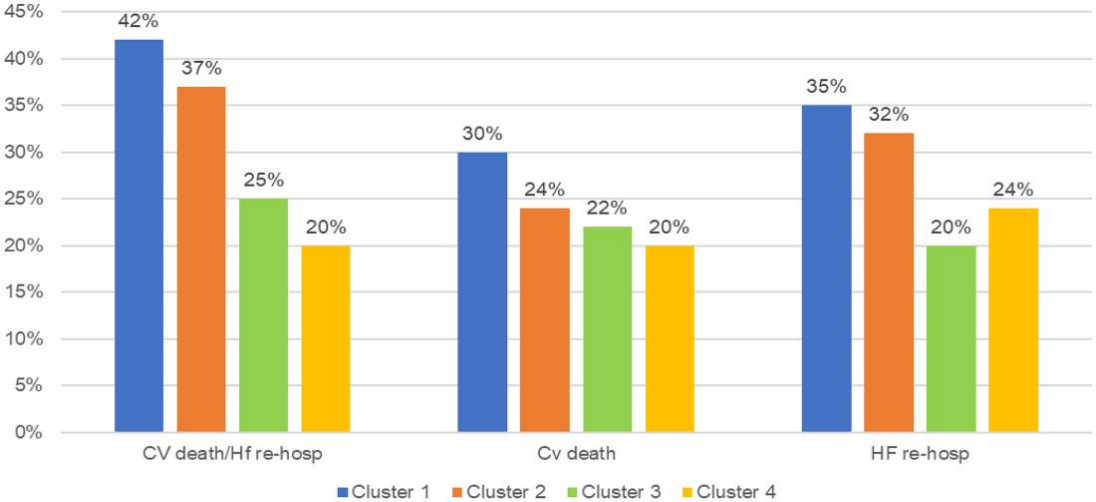
Incidence of primary and secondary endpoint according to clustering. CV, cardiovascular; HF, heart failure.

Patients’ characteristics divided per cluster (*a priori*) are reported in *Table 1* and *Figures 1* and *2*. One hundred and seventy-six patients were included in Cluster 1 (21%), 233 (28%) in Cluster 2, 195 (24%) in Cluster 3, and 218 (27%) in Cluster 4. The composite primary endpoint occurred in 42, 37, 25, and 20% of patients within Clusters 1, 2, 3, and 4, respectively; cardiovascular death occurred in 30, 24, 22, and 20% of patients, whereas HF hospitalizations occurred in 35, 32, 20, and 24% of patients within each cluster, respectively (*Figure 3*).

**Table 1:** A priori data for clustering (all data are reported as absolute number and percentage or median and I and III inter-quartile range)

	Cluster 1 (*n* = 176, 21%)	Cluster 2 (*n* = 233, 28%)	Cluster 3 (*n* = 195, 24%)	Cluster4 (*n* = 218, 27%)
Age (years)	74 (65–79)	66.0 (59.0–73.0)	78.0 (72.0–83.0)	79.0 (73.0–84.0)
Female gender	49 (28%)	49 (21%)	105 (54%)	96 (44%)
BMI (kg/m^2^)	25 (22–28)	24.2 (21.3–26.0)	25.8 (23.5–27.3)	26.2 (23.4–29.4)
NYHA classes				
NYHA II	26 (15%)	36 (15.6%)	111 (57%)	15 (7%)
NYHA III	136 (77%)	140 (60%)	66 (34%)	172 (79%)
NYHA IV	12 (7%)	56 (24%)	18 (9%)	31 (14%)
Diabetes mellitus	74 (42%)	58 (25%)	55 (28%)	48 (22%)
Prior myocardial infarction	72 (41%)	114 (49.1%)	73 (37.4%)	50 (22.9%)
Paroxysmal AF	84 (48%)	37 (16%)	82 (42%)	39 (18%)
Persistent AF	9 (5%)	37 (16%)	14 (7%)	133 (61%)
Left ventricle end-diastolic volume/Body syrface area (BSA, mL/m^2^)	121 (107–159)	120 (107–147)	81 (66–107)	103 (77–107)
LVEF (%)	30 (25–35)	26 (24–31)	45 (35–55)	46 (35–55)
Tricuspid annular plane excursion (TAPSE, mm)	18 (15–20)	15 (13–18)	20(18–23)	18 (16–22)
Systolic pulmonary pressure (sPAP, mmHg)	45 (37–50)	52 (45–60)	45 (35–56)	50 (45–60)

Regarding a priori variables, compared with Clusters 3 and 4, patients in Clusters 1 and 2 had more dilated LVs [LV end-diastolic volume index 120.7 (107.2–159.0) mL/m^2^ and 120.2 (107.2–146.8) mL/m^2^ vs. 81.0 (66.2–107.2) mL/m^2^ and 102.6 (76.9–107.2) mL/m^2^, respectively], they were more frequently male (72 and 80% vs. 56 and 46%) and younger [74.0 (65.0–79.0) years and 66.0 (59.0–73.0) years vs. 79.0 (73.0–84.0) years and 78.0 (72.0–83.0) years] and had lower LVEF [30.0% (25.0–35.0) and 26.0% (24.0–31.0) vs. 45.0% (35.0–55.0) and 46.0% (35.0–55.0)]. The prevalence of permanent atrial fibrillation (AF) in Clusters 1–4 was 4.5, 15.6, 6.7, and 60.7%, respectively, whereas that of diabetes mellitus was 41.5, 24.9, 27.7, and 21.6%.

A posteriori variables are reported in *Tables 2–4*. Patients in Clusters 1 and 2 showed higher prevalence of coronary artery disease (51 and 60% vs. 40 and 30%, respectively), chronic kidney disease (67 and 58% vs. 46 and 56%, respectively) and Grade 3 diastolic dysfunction (48 and 64% vs. 20 and 32%) compared with patients in Clusters 3 and 4. No difference regarding right heart catheterization data was found between clusters of the 252 patients with these parameters available, except for a lower cardiac index in Cluster 2. To obtain a practical algorithm, we created two profiles of patients (*Graphical abstract*), namely a high-risk phenotype (Clusters 1 and 2), and a low-risk phenotype (Clusters 3 and 4). High-risk phenotype patients had larger LVs (>107 mL/m^2^), lower LVEF (<35%) and more prevalent ischaemic aetiology (51 to 60% vs. 30 to 40%) compared with low-risk phenotype patients. Moreover, within the high-risk group, patients with diabetes mellitus and with advanced age were at increased risk of experiencing the primary endpoint. Within the low-risk group, ischaemic aetiology increased the risk of cardiovascular death, while permanent AF amplified that of HF hospitalization.

**Table 2:** *A posteriori* baseline data (all data are reported as absolute number and percentage or median and I and III inter-quartile range)

	Cluster 1 (*n* = 176, 21%)	Cluster 2 (*n* = 233, 28%)	Cluster 3 (*n* = 195, 24%)	Cluster4 (*n* = 218, 27%)
Hypertension	111 (63%)	121 (52%)	142 (73%)	159 (73%)
Hyperlipidaemia	88 (50%)	110 (47%)	98 (50%)	89 (41%)
Smoking				
Previous	44 (25%)	70 (30%)	14 (7%)	28 (13%)
Active	26 (15%)	26 (11%)	4 (2%)	24 (11%)
Chronic obstructive pulmonary disease^a^	48 (27%)	47 (20%)	45 (23%)	72 (33%)
Peripheral artery disease (PAD)^b^	25 (14%)	28 (12%)	31 (16%)	37 (17%)
Prior stroke	14 (8%)	14 (6%)	29 (15%)	20 (9%)
Coronary artery disease^c^	90 (51%)	140 (60%)	78 (40%)	65 (30%)
Chronic kidney disease^d^	118 (67%)	135 (58%)	90 (46%)	122 (56%)
CRT^e^	77 (44%)	79 (34%)	55 (28%)	48 (22%)
Medical therapy before valvular intervention				
1. Beta blockers	150 (85%)	210 (90%)	158 (81%)	187 (86%)
2. Diuretics	167 (95%)	226 (97%)	174 (89%)	201 (92%)
3. ACE-inhibitors, angiotensin receptor blockers	111 (63%)	119 (51%)	103 (53%)	137 (63%)
4. Sacubitril/valsartan	26 (15%)	44 (19%)	47 (24%)	17 (8%)
5. Mineralocorticoid receptor antagonist	118 (67%)	163 (70%)	70 (36%)	153 (70%)

^a^Chronic obstructive pulmonary disease.

^b^Peripheral artery disease.

^c^Defined as previous myocardial infarction/percutaneous coronary intervention/surgical revascularization.

^d^Defined as mL/min/1.73 m^2^.

^e^Cardiac resynchronization therapy.

**Table 3:** *A posteriori* echocardiography data (all data are reported as absolute number and percentage or median, and I and III inter-quartile range)

	Cluster 1 (*n* = 176, 21%)	Cluster 2 (*n* = 233, 28%)	Cluster 3 (*n* = 195, 24%)	Cluster 4 (*n* = 218, 27%)
Left ventricle end-diastolic diameter (LVEDD, mm)	68 (63–76)	70 (62–75)	55 (51–61)	58 (50–68)
Left ventricle end-diastolic volume (LVEDV, mL)	208 (173–261)	205 (177–242)	124 (100–154)	142 (105–182)
Left atrial volume/BSA (mL/m^2^)	90 (58–117)	84 (62–110)	48 (31–83)	65 (49–92)
Mitral regurgitation				
Moderate	30 (17%)	37 (16%)	49 (25%)	26 (12%)
Severe	141 (80%)	191 (82%)	146 (75%)	190 (87%)
Diastolic dysfunction				
Grade I	28 (16%)	21 (9%)	78 (40%)	89 (41%)
Grade II	63 (36%)	61 (26%)	78 (40%)	59 (27%)
Grade III	84 (48%)	149 (64%)	39 (20%)	70 (32%)
Right ventricle diameter (mm)	38 (32–41)	39 (35–45)	36 (33–40)	40 (35–44)

**Table 4:** *A posteriori* right heart catheterization data (all data are reported as median and I and III inter-quartile range)

	Cluster 1 (*n* = 52, 21%)	Cluster 2 (*n* = 103, 41%)	Cluster 3 (*n* = 34, 13%)	Cluster 4 (*n* = 63, 25%)
Pulmonary arterial pressure (mmHg)				
1. Systolic	47 (34–58)	47 (36–64)	39 (34–55)	46 (35–61)
2. Diastolic	19 (13–24)	21 (17–27)	17 (14–22)	20 (14–26)
3. Mean	31 (20–38)	30 (23–40)	28 (22–36)	30 (22–39)
Wedge pressure (mmHg)	21 (11–30)	21 (15–28)	18 (13–23)	20 (14–25)
V-wave (mmHg)	24 (14–44)	27 (16–40)	25 (10–28)	25 (17–29)
Transpulmonary mean gradient (mmHg)	9 (7–12)	10 (7–13)	10 (6–14)	9 (6–16)
Right atrial pressure (mmHg)	6 (5–9)	6 (4–10)	6 (5–10)	9 (6–10)
Cardiac output (L/min/m^2^)	3 (3–4)	3 (3–4)	4 (3–4)	4 (3–4)
Cardiac index (L/min/m^2^)	2 (1–2)	2 (1–2)	2 (2–3)	2 (2–3)
Pulmonary vascular resistance (WU m^2^)	2 (2–3)	2 (1–4)	2 (1–3)	2 (1–3)
Pulmonary artery pulsatility index	4 (3–7)	5 (3–7)	3 (2–8)	3 (2–5)

Additional analysis comparing outcomes across clusters before and after 2015 showed similar trends in the cohort 2009–15 (primary endpoint 66.2 vs.45.2% vs. 40.7 vs. 35.6%) and in the cohort 2016–22 (26 vs. 29% vs. 17.6 vs. 11.8%).

### Clustering on the Mitrascore cohort

Baseline and echocardiographic data are reported in Supplementary material online, *Tables S4* and *S5*. Two hundred and sixty (23%) patients were included in Cluster 1, 360 (32%) in Cluster 2, 234 (21%) in Cluster 3, and 265 (24%) in Cluster 4. Patients in high-risk clusters were more frequently male, with higher rates of ischaemic heart disease, younger, with more dilated LVs and lower LVEF. Patients in the low-risk clusters were older, and with higher prevalence of AF.

**Figure 4:**
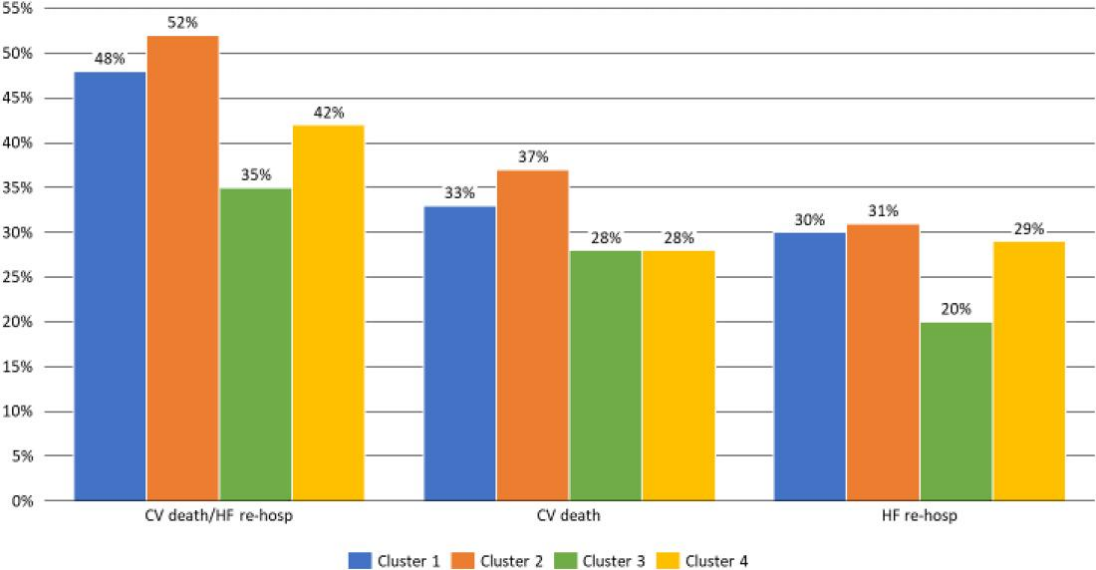
Incidence of events according to clustering in Mitrascore dataset. CV, cardiovascular; HF, heart failure.

After a median follow-up of 1.6 years (inter quartile range [IQR]: 0.7–3.1) the incidence of the primary endpoint was 48, 52, 35, and 42% in Clusters 1, 2, 3 and 4, respectively, while that of cardiovascular death was 33, 37, 28, and 28% and that of re-hospitalization for HF was 30, 31 20, and 29%, respectively (*Figure 4*).

### Comparison with the Mitrascore

**Figure 5:**
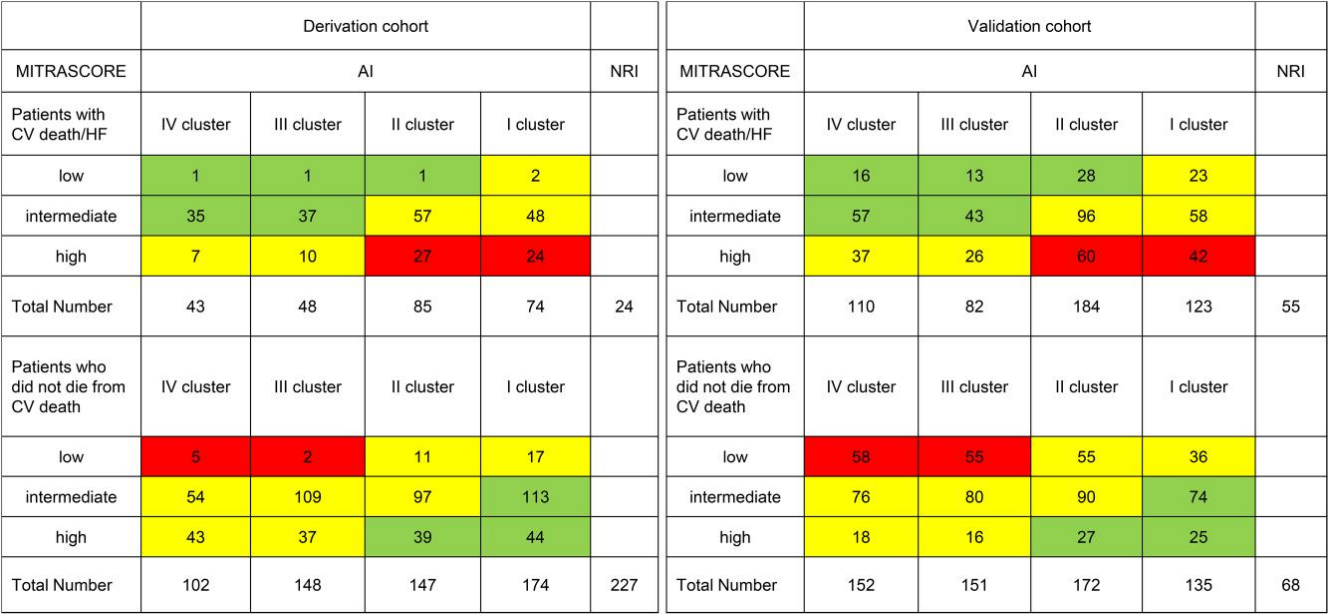
Net reclassification index on the derivation cohort on the left and on the validation on the right (green means improvement in classification, yellow no difference, red worsening with AI model set as reference). NRI, net reclassification index.

The Mitrascore performed accurately in the derivation cohort of the MITRA-AI project. The incidence of the primary endpoint according to the class of risk of Mitrascore ranged from 12.5 to 32.2 to 29.4%, while rates of death were respectively 16, 23, and 20% and those of HF re-hospitalization 12, 30, and 25%. Net reclassification indexes between clustering and Mitrascore were performed in both derivation and validation cohorts (NRI) and reported in *Figure 5*. In the derivation cohort clustering achieved a NRI of 27%, while in the validation cohort the NRI was 7%.

### Clustering on the cohort of patients with functional mitral regurgitation treated medically

Clustering assignment was performed in 207 patients with moderate-to-severe functional MR treated with medical therapy. About 18% of patients were included in Cluster 1, 46% in Cluster 2, 5% in Cluster 3, and 31% in Cluster 4. Baseline features among clusters varied consistently with those described before (see Supplementary material online, *Tables S6* and *S7*). Patients in the first two clusters were younger, but with higher rates of ischaemic heart disease: those in the low-risk clusters were older, with less dilated left ventricles and with higher prevalence of AF.

**Figure 6:**
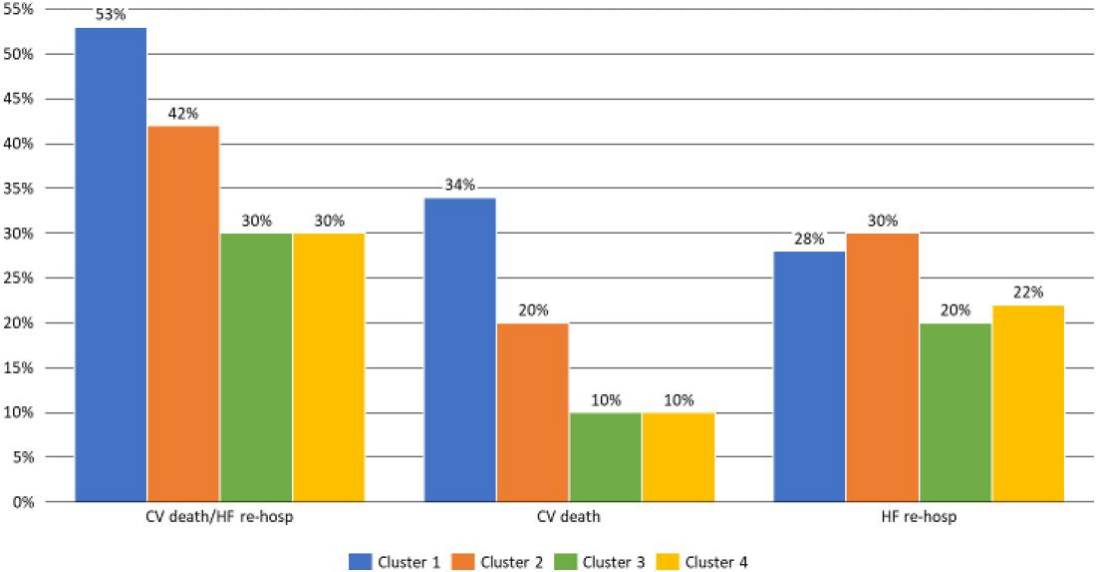
Incidence of events according to clustering in optimal medical therapy dataset. CV, cardiovascular; HF, heart failure.

The primary endpoint occurred in 53% of patients in Cluster 1, in 42% in Cluster 2, and in 30% in both low-risk clusters. Rates of cardiovascular death and HF re-hospitalizations varied consistently in Clusters 1–4 (34, 20, 10, and 10%, respectively and 28, 30, 20, and 22%, respectively) (*Figure 6*).

## Discussion

The aim of this study was to use ML to phenotype patients undergoing TEER. By analysing in a hidden and non-linear way the mitral valve pathology together with clinical, echocardiographic, and haemodynamic characteristics, four clusters of patients with increasing risk of cardiovascular death or HF hospitalizations were detected, which may be combined into a high-risk phenotype (Clusters 1 and 2) and a low-risk phenotype (Clusters 3 and 4). Compared with the low-risk phenotype, the high-risk phenotype showed a higher prevalence of male sex and prior acute myocardial infarction, greater LV volumes, and lower LVEF. Within the high-risk phenotype, Cluster 1 patients were older, with a higher prevalence of diabetes mellitus and chronic kidney disease compared with Cluster 2 patients; conversely, within the low-risk phenotype, Cluster 4 patients had larger left atrial volume index and higher prevalence of permanent AF than Cluster 3 patients. This approach showed good performance in two different validation cohorts consisting of patients undergoing MitraClip implantation and patients treated medically.

Prior studies analysing the impact of mitral valve disease on clinical outcomes usually imply a direct causal relationship between valvular defects and the development of co-occurring cardiac pathologies in a linear fashion, potentially overlooking the influence of exacerbating comorbidities.^18^ Our phenotyping approach departs from traditional, divisive classification systems based on preconceived models by adopting a ML clustering method. This novel approach offers a fresh perspective on the intricate, non-linear accumulation of extra-mitral valve cardiac damage, which can arise from diverse pathophysiologies, disease progression stages, and the impact of comorbidities.

It comes as no surprise that greater LV dilatation and poorer LVEF were associated with an ominous prognosis. Both LV dilatation and dysfunction characterize a more advanced disease state and inherently depict a population at higher risk of cardiovascular events.^19^ Moreover, these features portray the so-called ‘proportionate’ FMR, which would rather benefit from reversal of LV remodelling and reduction of LV volumes than the direct reduction of the effective regurgitant orifice area.^15^

Coronary artery disease was more common in the high-risk phenotype; this finding is in agreement with the results of a recent meta-analysis showing no benefit of the MitraClip procedure compared with medical therapy alone in reducing mortality and repeated hospitalizations among patients with ischaemic MR.^20,21^ Brouwer *et al*.^22^ described how reverse remodelling, occurring in about half of patients undergoing TEER, was associated with lower mortality than adverse remodelling (27 vs. 67%), and a large multicentre registry identified female gender, non-ischaemic aetiology of MR, and smaller LV end-diastolic diameter as independent predictors of reverse remodelling.^23^ Therefore, factors associated with positive or adverse LV remodelling might represent a critical factor in determining long-term results after TEER.

Moreover, acute ischaemic events following MitraClip implantation were described to occur more frequently in patients with pre-existing ischaemic heart disease.^24^ Finally, patients with coronary artery disease who have moderate-to-severe and ‘proportionate’ FMR require careful study and selection before undergoing MitraClip implantation due to their residual high risk of cardiovascular events following TEER. Future studies are warranted to assess whether these patients may respond better to interventions directed at the LV rather than the mitral valve.

Notably, the high-risk phenotype was burdened by a higher risk of HF hospitalizations compared with the low-risk phenotype (35 and 32% vs. 20 and 24%), but only Cluster 1 was associated with a higher risk of cardiovascular death at 1 year follow-up, likely due to the older age and higher prevalence of diabetes mellitus and impaired kidney function of this cluster within the high-risk group.^25–27^ Our results support those of the MitraScore, which was recently validated to predict follow-up mortality after TEER.^28^

Cluster 4, representing the lowest-risk group, showed some distinctive features. First, history of coronary artery disease and prior percutaneous coronary intervention were less prevalent in Cluster 4 than in Clusters 1 to 3. Although LVEF was mildly reduced in both Clusters 3 and 4, severe diastolic dysfunction was less common in Cluster 4 than in all the other clusters, probably representing a less advance LV disease. Importantly, in the presence of a non-dilated LV, permanent AF was much more prevalent in Cluster 4 than in other clusters, and within the low-risk phenotype itself the left atrium was larger in Cluster 4 than Cluster 3; these characteristics may depict a condition recently recognized as atrial functional MR, in which atrial remodelling induces mitral annulus dilatation and flattening. Functional mitral regurgitation undergoing TEER has been associated with a worse prognosis than degenerative MR,^11^ but atrial FMR overall showed better clinical outcomes compared with ventricular FMR^26^ and the latter holds true also for patients treated surgically.^29–31^

Despite right ventricular function was described to yield an important prognostic role in patients undergoing TEER, unsupervised phenotyping did not stratify patients by right ventricular function in the present analysis. This finding might be explained by the overall preserved right ventricular function of the included population, with only rare cases of severe right ventricular disease insufficient to configure different prognostic classes within this model. Nevertheless, this finding deserves specific studies focused on this issue and cannot be considered conclusive.

Among patients with moderate-to-severe FMR treated medically, clustering analysis by means of ML discriminated high-risk clusters, consisting of younger patients with higher rates of ischaemic heart disease and chronic kidney disease, and low-risk clusters, whose patients had less dilated LVs with more frequent AF. This ML phenotypization among patients treated medically overlaps with that of patients undergoing TEER, and accurately describes different clusters with different risks of cardiac events among FMR patients treated conservatively. These results, associated with morphomic and functional data on secondary mitral valve components derived from a prior FMR clustering analysis,^32^ may help enhance risk prediction in patients undergoing MitraClip intervention. This further emphasizes the potential role of ML analysis in predicting cardiovascular events among FMR patients treated conservatively or invasively, always bearing in mind that clinicians, in addition to striving for accurate mortality predictions, should try to comprehend these predictions, put them into individual contexts and articulate to patients the underlying factors that contribute to these predictions in a simple manner.

### Limitations

This study is based on an observational registry and carries all the inherent limitations of such studies. In preparing the dataset for statistical analyses, variables with more than 35% of missing values were excluded. This relatively high percentage was accepted in order to include some important variables such as body mass index and tricuspid annular plane systolic excursion. Several factors are missing from the clustering model; however, it was decided to select only the variables most clinically and pathophysiologically related to the primary outcome to reduce confounding data and to facilitate the development of a user-friendly risk-prediction tool. Regarding data not collected, comparison with the MITRALITY score was not feasible, due to some missing data (urea).^33^ Finally, an important limitation lies in the ML model itself: allowing to capture complex and non-linear relationships it may also derive non causal connection, and from this point of view the present analyses are merely descriptive, without inferential aim. Due to the lack of MR quantitative data, the ‘proportionality’ framework could not be explored.

## Conclusions

We proposed a novel ML analysis to identify meaningful clinical phenotypic presentations in FMR patients undergoing TEER. We identified four clusters with increasing risk of cardiovascular death or HF hospitalization which may be combined into a high-risk (Clusters 1 and 2) and a low-risk phenotype (Clusters 3 and 4). Future studies are needed to assess how these phenotypes can be handled to identify those patients who could derive most benefit from TEER for FMR.

## Supplementary Material

ztaf006_Supplementary_Data

## Data Availability

The present data may be available after request to investigators, according to kind of request.
