# Supplementary material for: Machine-learning phenotyping of patients with functional mitral regurgitation undergoing transcatheter edge-to-edge repair: the MITRA-AI study
Source: Eur Heart J Digit Health. 2025 Feb 13;6(3):340–9. doi: 10.1093/ehjdh/ztaf006 (PMC12088727; doi:10.1093/ehjdh/ztaf006)
Supplement: ztaf006_Supplementary_Data [file ztaf006_supplementary_data.docx]

**Supplementary Appendix**

This appendix has been provided by the authors to give readers additional information about their work.

**SUPPLEMENTARY APPENDIX**

**Table of Contents**

- Participating Centers .................................................................................................................................3

- Contributing authors……………………………………………………………………………………………….4

- Figure S1. Percentages of a-priori variables without missing values ….....................................................5

- Figure S2. Variables distribution stratified based on the patients’ status (alive vs dead)...........................6

- Figure S3. Selection of the optimal number of clusters based on the elbow method (blue curve) and a composite metric based on the silhouette coefficient score (orange curve) .…………….............................7

- Figure S4. Distribution of continuous variables according to clusters (IQR) ………………………………8

- Figure S5. Selection of the optimal number of clusters based on the elbow method (blue curve) and a composite metric based on the silhouette coefficient score (orange curve) using MICE 9

-- Table S1 Baseline features of included patients (all data are reported as percentage or median and I and III interquartile range)…………………………………………………………………………………………..10

- Table S2. Echocardiography data (all data are reported as percentage or median and I and III interquartile range)………………………………………………………………………………………………….11

- Table S3. Right Heart Catheterization data (all data are reported as percentage or median and I and III interquartile range)………………………………………………………………………………………………….12

- Table S4. Baseline features of included patients according to clustering on Mitrascore dataset (all data are reported as percentage or median and I and III interquartile range)………………………..……………13

- Table S5. Echocardiography data of included patients according to clustering on Mitrascore dataset (all data are reported as percentage or median and I and III interquartile range)……………………………….15

- Table S6. Baseline features of included patients according to clustering on patients treated medically (all data are reported as percentage or median and I and III interquartile range)……………………………….16

- Table S7.Echocardiography data of included patients according to clustering on patients treated medically (all data are reported as percentage or median and I and III interquartile range)……………..18

- Table S8. Pearson correlation coefficient between BMI and the other variables included in the clustering model…………………………………………………………………………………..……………..18

- Table S9. Pearson correlation coefficient between TAPSE and the other variables included in the clustering model…………………………………………………………………………………..……………..19

**- Sensitivity analysis for the imputation method…………………………………………………………….. 20**

Participating Centers

**Leading Study Center**

Division of Cardiology, Cardiovascular and Thoracic Department, "Citta della Salute e della Scienza" Hospital, Turin, Italy; Department of Medical Sciences, University of Turin, Turin, Italy – 122 patients

**Participating Study Centers**

Cardiovascular Interventional Operative Unit, Presidio Ospedaliero Pineta Grande, Castel Volturno, Caserta, Italy; Operative Unit of Hemodynamics, Casa di Salute Santa Lucia, San Giuseppe Vesuviano, Naples, Italy – 165 patients

Cardiology Unit, DICATOV-Cardiothoracic and Vascular Department, IRCCS San Martino Hospital, Genoa, Italy – 11 patients

Cardiac Intensive Care Unit and De Gasperis Cardio Center, ASST Grande Ospedale Metropolitano Niguarda, Milan, Italy – 79 patients

Dipartimento Universitario di Medicina Traslazionale, Università Piemonte Orientale, Azienda Ospedaliero-Universitaria Maggiore della Carità di Novara, Novara, Italy - 7 patients

Cardio Thoracic and Vascular Department, Azienda Ospedaliero-Universitaria Pisana, Pisa, Italy – 167 patients

Cardiology Department, Rabin Medical Center, Petah Tikva, Israel; Sackler Faculty of Medicine, Tel Aviv University, Tel Aviv, Israel – 76 patients

Department of Medicine, 'Tor Vergata' University of Rome, Rome, Italy – 45 patients

Department of Cardiology, University Heart Center, University Hospital Zurich, Zurich, Switzerland – 119 patients

Division of Cardiology, Fondazione IRCCS Policlinico San Matteo Foundation – 33 patients

Cardiology and Cardiac Catheterization Laboratory, Cardio-Thoracic Department, Civil Hospitals, Department of Medical and Surgical Specialties, Radiological Sciences, and Public Health, University of Brescia, Brescia, Italy - validation cohort

Cardiology Department, University Hospital Álvaro Cunqueiro, Estrada de Clara Campoamor, 341, 36213, Vigo, Pontevedra, Spain - validation cohort

**Contributing authors**

Guglielmo Gallone MD, Francesco Bruno MD, Matteo Bellettini MD, Alessandro Vairo MD, Vittoria Scavino MD, Division of Cardiology, Cardiovascular and Thoracic Department, "Citta della Salute e della Scienza" Hospital, Turin, Italy; Department of Medical Sciences, University of Turin, Turin, Italy

Alberto Morello MD, Cardiovascular Interventional Operative Unit, Presidio Ospedaliero Pineta Grande, Castel Volturno, Caserta, Italy; Operative Unit of Hemodynamics, Casa di Salute Santa Lucia, San Giuseppe Vesuviano, Naples, Italy

Marco Mennuni MD, Dipartimento Universitario di Medicina Traslazionale, Università Piemonte Orientale, Azienda Ospedaliero-Universitaria Maggiore della Carità di Novara, Novara, Italy

Massimo Sangiorgi Division of Cardiology, Department of Systems Medicine, Tor Vergata University, 00133 Rome, Italy

Xavier Freixa Hospital Clinic, Barcelona, Spain.

Dabit Arzamendi Interventional Cardiology Unit, Hospital Sant Pau i Santa Creu, Barcelona, Spain

Tomas Benito-González Complejo Asistencial Universitario de León, León, Spai

Isaac Pascual Interventional Cardiology Unit, Hospital Universitario Central de Asturias, Oviedo, Spain

Luis Nombela-Franco Cardiovascular Institute, Hospital Clinico San Carlos, IdISSC, Madrid, Spain

Josep Rodes-Cabau Cardiology Department, Quebec Heart and Lung Institute, Laval University, Quebec City, Quebec, Canada

Mony Shuvy Heart Institute, Hadassah-Hebrew University Medical Center, Jerusalem, Israel

Antonio Portolés-Hernández Cardiology Department, Hospital Universitario Puerta de Hierro, Majadahonda, Madrid

Cosmo Godino Clinical Cardiology Unit, Faculty of Medicine, IRCCS San Raffaele Scientific Institute, Milan, Italy.

Luca Testa Department of Cardiology, IRCCS Policlinico San Donato, San Donato Milanese, Milan, Italy.

Carmelo Grasso Division of Cardiology, Centro Alte Specialità e Trapianti (CAST), Azienda Ospedaliero-Universitaria Policlinico-Vittorio Emanuele, University of Catania, Catania, Italy.

**Figure S1. Percentages of a-priori variables without missing values**
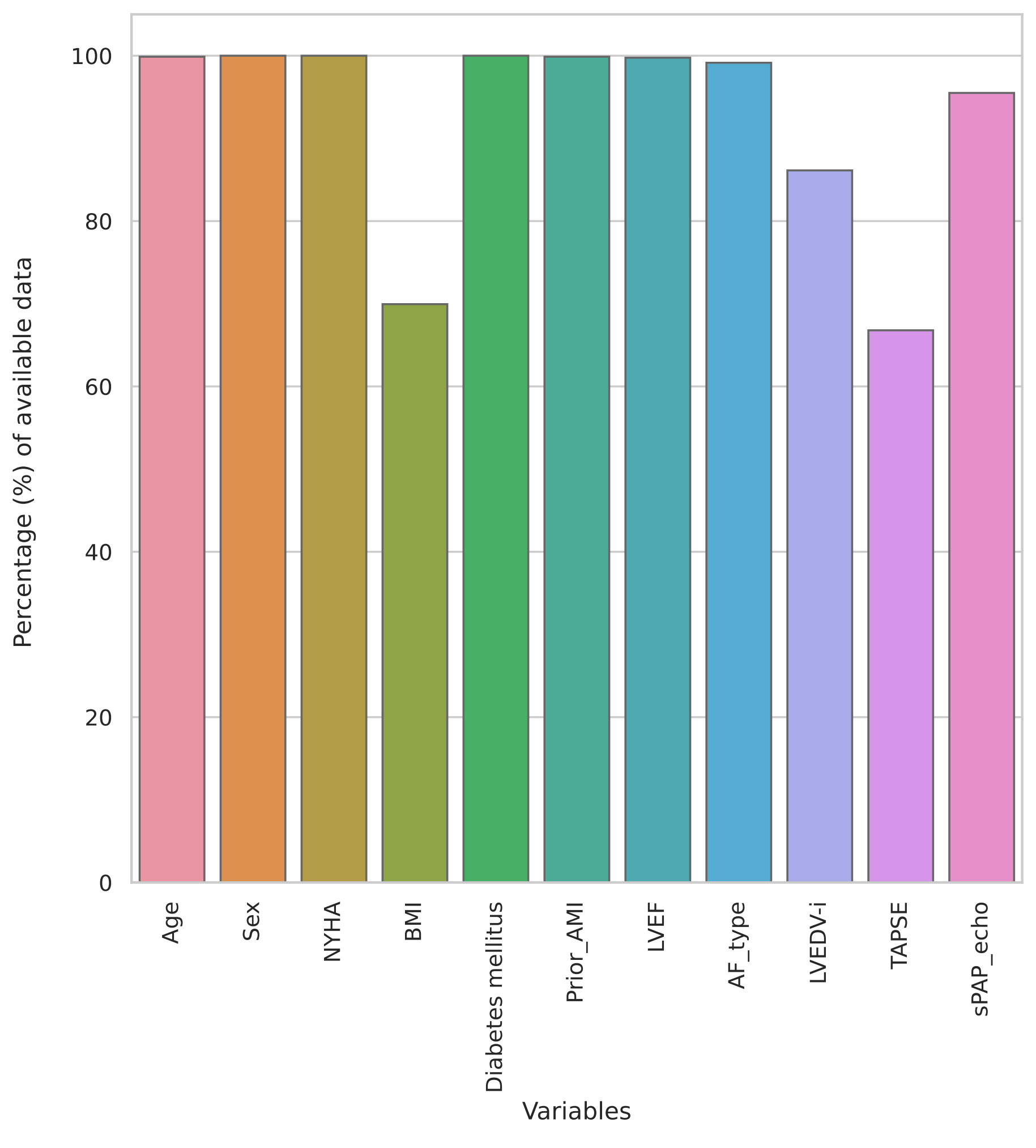


**Figure S2. Variables distribution stratified based on the patients’ status (alive vs dead)**
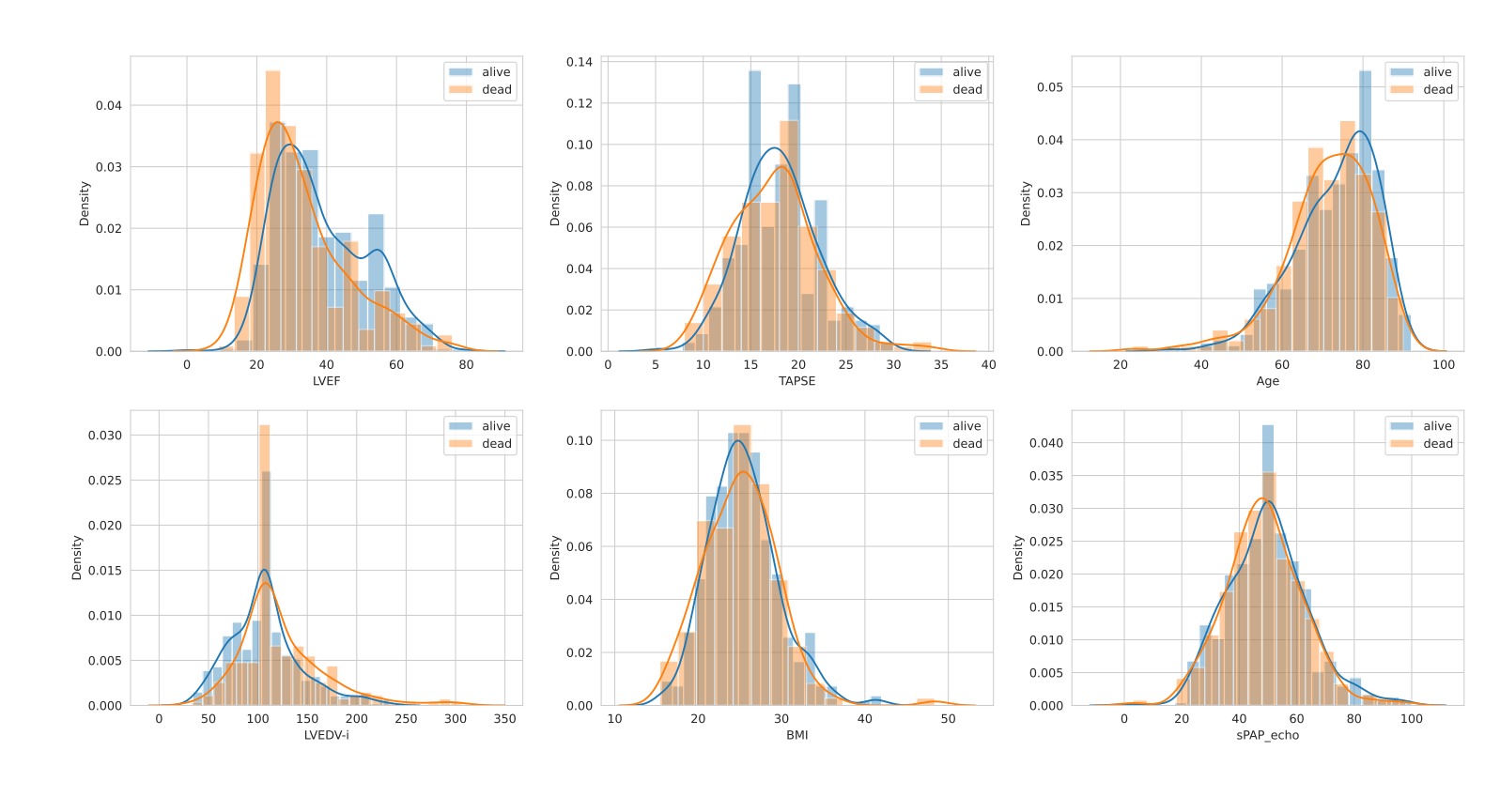


BMI = body mass index; LVEDVi = left ventricle end diastolic volume index; LVEF = left ventricular ejection fraction; sPAP = systolic pulmonary artery pressure; TAPSE = tricuspid annular plane excursion

**Figure S3. Selection of the optimal number of clusters based on the elbow method (*blue curve*) and a composite metric based on the silhouette coefficient score (*orange curve*).**


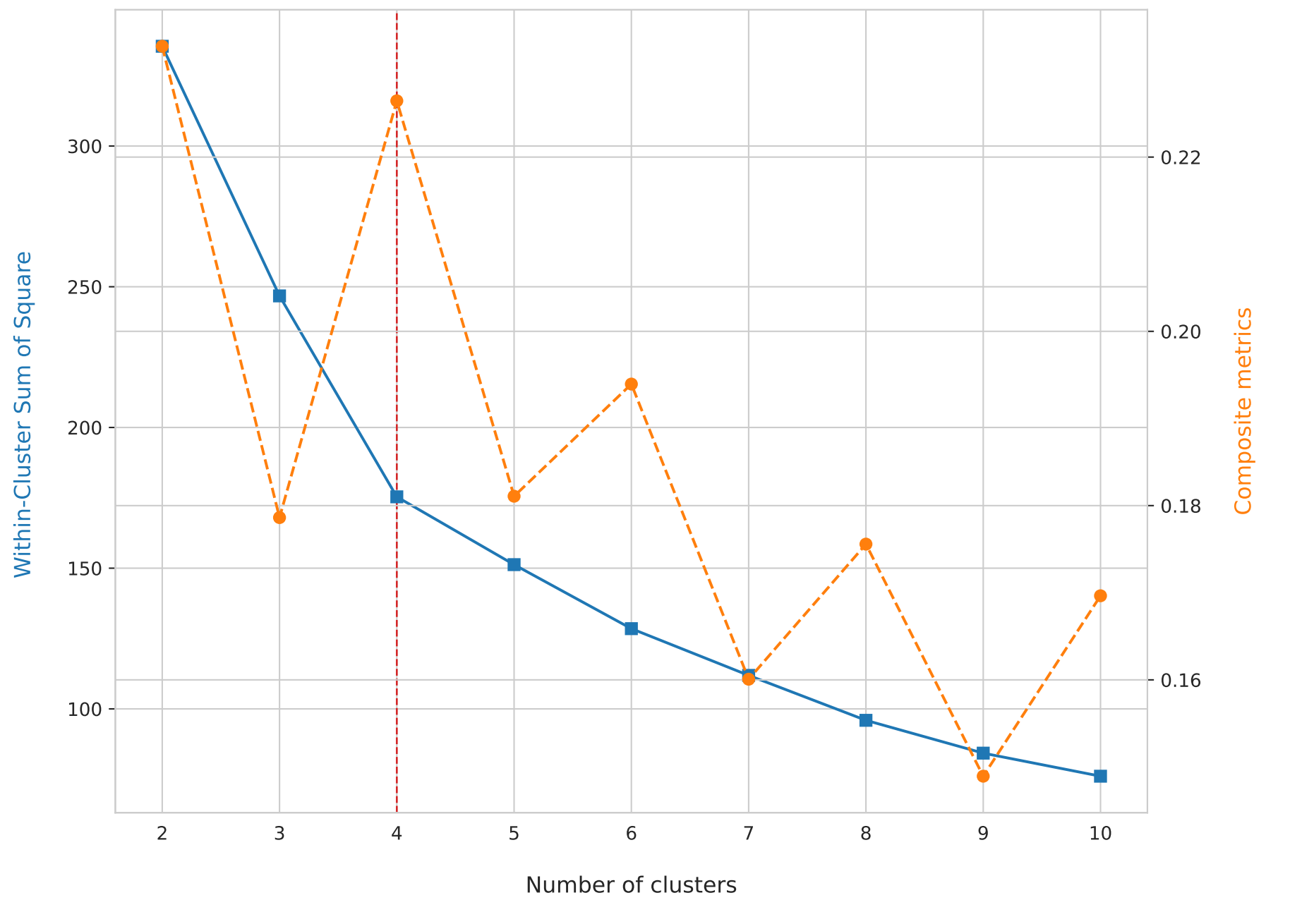


**Figure S4. Distribution of continuous variables according to clusters (IQR)**


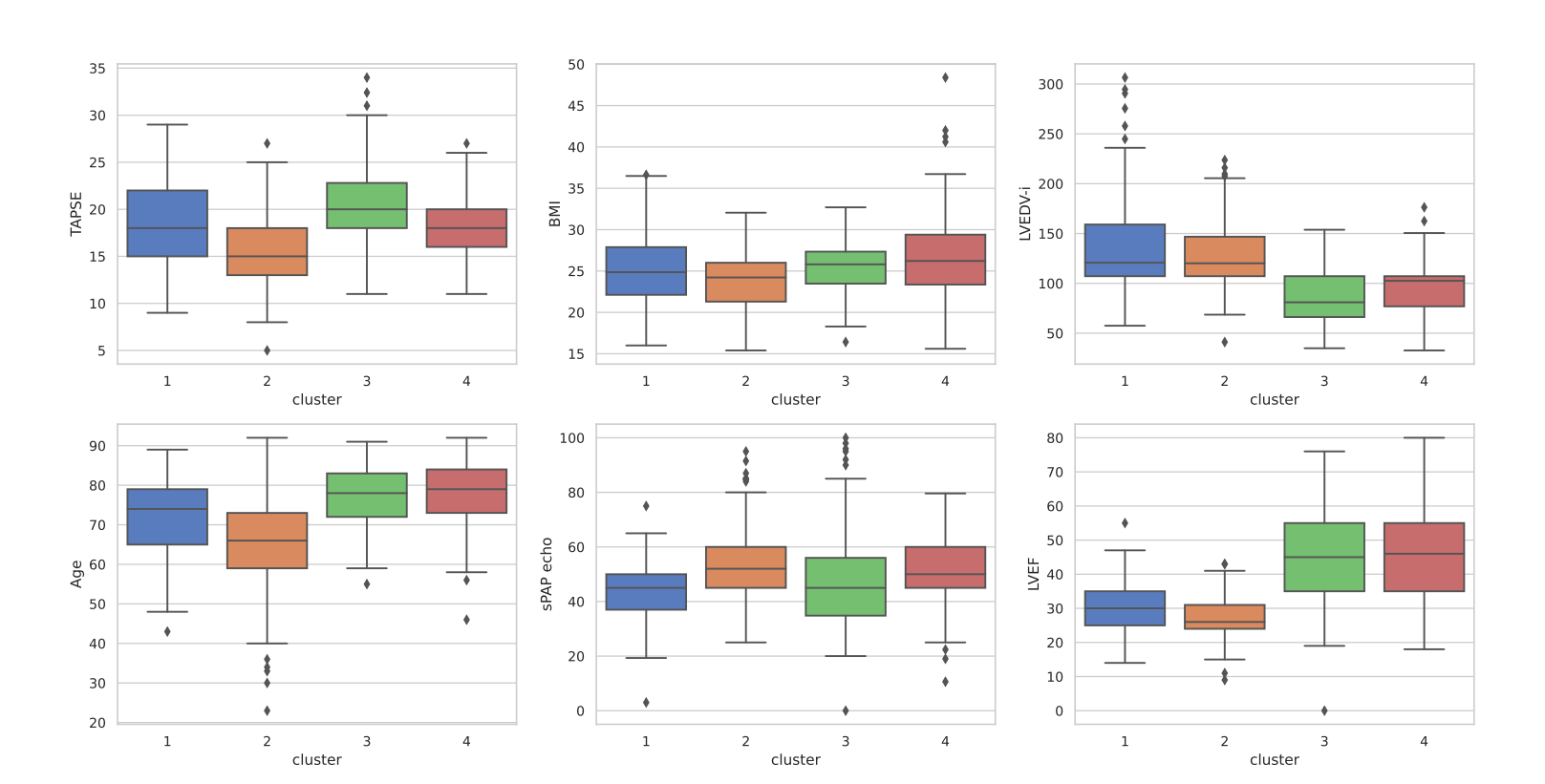


See Figure S2 for abbreviations

**Figure S5. Selection of the optimal number of clusters based on the elbow method (*blue curve*) and a composite metric based on the silhouette coefficient score (*orange curve*) utilizing MICE imputation method.**


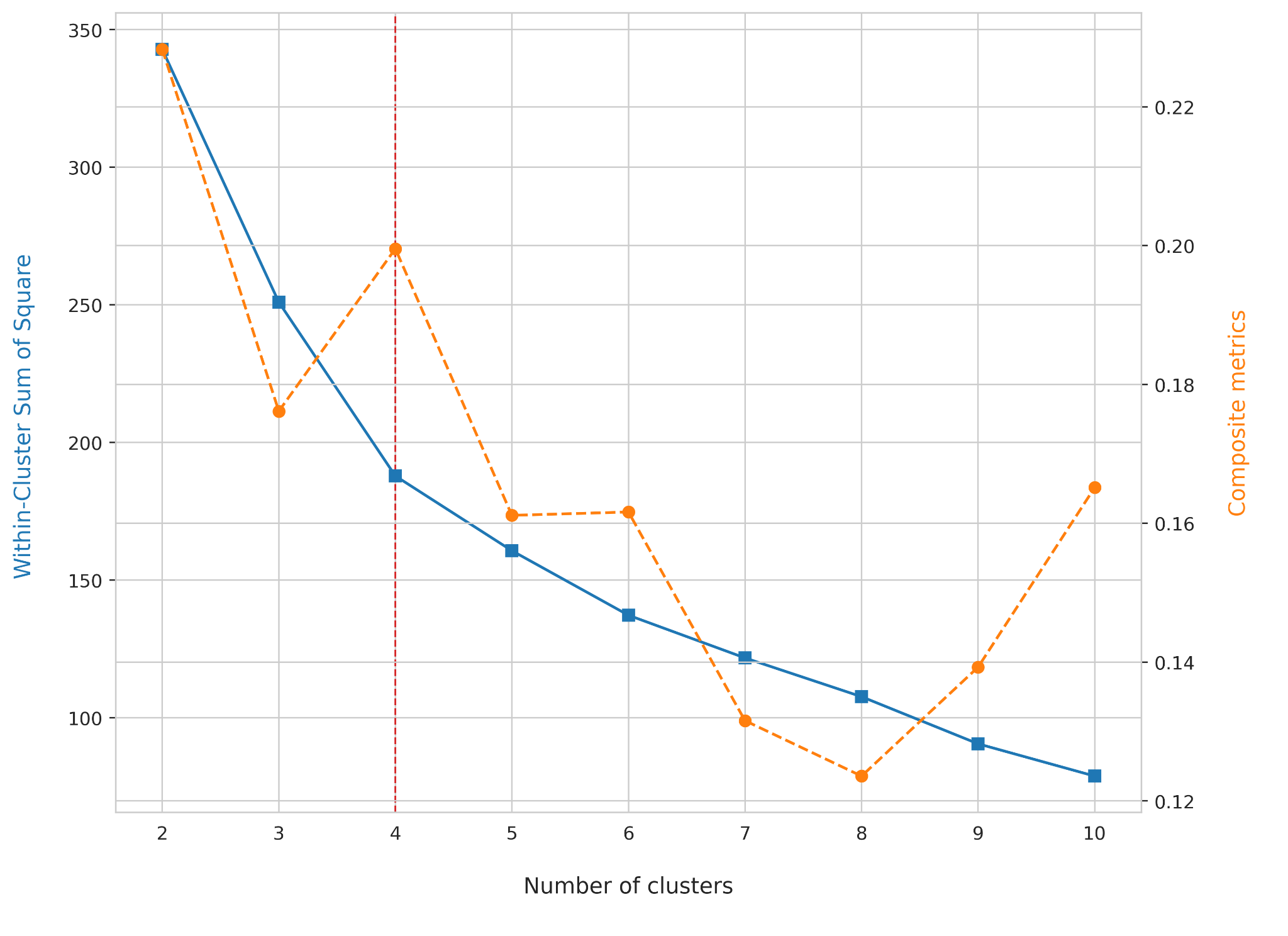


**Table S1. Baseline features of included patients** (all data are reported as absolute number and percentage or median and interquartile range)

|  | Patients experiencing CV death/HF hospitalizations  (n 250, 30%) | Patients not experiencing CV death/HF hospitalizations  (n 572, 70%) | P value |
| --- | --- | --- | --- |
| Age (years) | 72 (68-75) | 73 (68-76) | 0.025 |
| Female gender | 79 (32%) | 218 (38%) | 0.072 |
| BMI (kg/m^2^) | 25 (19-27) | 25 (22-28) | 0.076 |
| Hypertension | 149 (60%) | 384 (67%) | 0.032 |
| Hyperlipidemia | 95 (40%) | 237 (51%) | 0.009 |
| Smoking  Previous  Active | 21 (7%)  30 (28%) | 17 (16%)  48 (17%) | 0.002 |
| Diabetes mellitus | 76 (30%) | 156 (27%) | 0.359 |
| COPD* | 61 (25%) | 146 (26%) | 0.710 |
| PAD** | 35 (21%) | 38 (11%) | 0.004 |
| Prior stroke | 11 (6%) | 39 (11%) | 0.071 |
| CAD*** | 124 (50%) | 214 (37%) | 0.070 |
| Atrial fibrillation   - paroxysmal - permanent | 71 (29%)  50 (20%) | 171 (30%)  137 (24%) | 0.275 |
| Chronic Kidney Disease**** | 63 (69%) | 125 (51%) | 0.005 |
| NYHA   - II - III - IV | 38 (16%)  161 (64%)  51 (20%) | 150 (27%)  354 (62%)  68 (12%) | <0.001 |
| CRT***** | 60 (39%) | 87 (32%) | 0.161 |
| Medical therapy before valvular intervention   - Beta blockers - Diuretics - ACE-inhibitors/   Angiotensin receptor blockers   - Valsartan/sacubitril - [Mineralocorticoid receptor antagonist](https://www.ncbi.nlm.nih.gov/pmc/articles/PMC5801434/) | 152 (84%)  243 (98%)  105 (59%)  19 (12%)  110 (68%) | 420 (86%)  518 (91%)  159 (56%)  53 (19%)  178 (57%) | 0.534  0.001  0.456  0.059  0.013 |

*Chronic Obstructive Pulmonary Disease; **Peripheral Artery Disease; *** defined as previous MI/PCI/CABG; ****defined as mL/min/1.73m^2^ ;**** Cardiac Resynchronization therapy

**Table S2. Echocardiography data** (all data are reported as absolute number and percentage or median and I and III interquartile range)

|  | Patients experiencing CV death/HF hospitalizations  (n 250, 30%) | Patients not experiencing CV death/HF hospitalizations  (n 572, 70%) | P value |
| --- | --- | --- | --- |
| Left ventricle ejection fraction (%) | 33 (31-34) | 38 (37-39) | <0.001 |
| Left ventricle end diastolic diameter (LVEDD,mm) | 66 (65-68) | 64 (63-66) | 0.219 |
| Left ventricle end diastolic volume (LVEDV, ml) | 210 (198-223) | 175 (167-180) | <0.001 |
| Left ventricle end diastolic volume/BSA (ml/m^2^) | 130 (122-138) | 107 (103-111) | <0.001 |
| Mitral regurgitation   - Moderate - Severe | 54 (22%)  188 (75%) | 87 (15%)  473 (82%) | 0.009 |
| Diastolic dysfunction   - Grade 1 - Grade 2 - Grade 3 | 5 (8%)  14 (22%)  43 (68%) | 36 (28%)  45 (35%)  48 (37%) | <0.001 |
| Right ventricle diameter (mm)* | 38 (36-40) | 38 (37-39) | 0.965 |
| Tricuspid annular plane excursion (TAPSE, mm) | 17 (16-18) | 18 (17-18) | 0.084 |
| Systolic Pulmonary pressure (sPAP mmHg) | 49 (47-50) | 49 (48-51) | 0.255 |

*four chambers

**Table S3. Right heart catheterization data (all data are reported as absolute number and percentage or median and I and III interquartile range).**

|  | Patients experiencing CV death/HF hospitalizations (109, 43%) | Patients not experiencing CV death/HF hospitalizations (142, 57%) | P value |
| --- | --- | --- | --- |
| Pulmonary arterial pressure (mmHg)   1. Sistolic 2. Diastolic 3. Mean | 48 (45-52)  21 (20-23)  31 (29-33) | 47(44-50)  20 (18-21)  31 (29-32) | 0.122  0.456  0.671 |
| Wedge Pressure (mmHg)  V-wave (mmHg) | 20 (19-22)  28 (22-33) | 20 (19-21)  25 (21-30) | 0.891  0.671 |
| Transpulmonary mean gradient (mmHg) | 10 (9-12) | 11 (10-12) | 0.876 |
| Right atrial pressure (mmHg) | 8 (7-9) | 7 (6-8) | 0.451 |
| Cardiac output (L/min/m2)  Cardiac Index (L/min/m2) | 3 (3-4)  2 (1-3) | 3 (3-4)  2 (1-2) | 0.681 |
| Pulmonary Vascular Resistance (WU m2) | 22 (17-28) | 16 (12-20) | 0.059 |
| [Pulmonary artery pulsatility index](https://onlinelibrary.wiley.com/doi/full/10.1002/ejhf.1679) | 4 (3-5) | 5 (4-6) | 0.143 |

**Table S4. Baseline characteristics of the Mitrascore cohort** (all data are reported as absolute number and percentage or mean ± standard deviation)

|  | **Cluster 1 (n=260, 23%)** | **Cluster 2 (n=360, 32%)** | **Cluster 3 (n=234, 21%)** | **Cluster4 (n=265, 24%)** |
| --- | --- | --- | --- | --- |
| Age (years old) | 72±9 | 67±10 | 79±7 | 79±7 |
| Female gender | 78 (30%) | 122 (34%) | 96 (41%) | 106 (40%) |
| BMI (kg/m^2^) | 26±4 | 25±4 | 26±4 | 27±6 |
| NYHA classes |  | | | |
| NYHA II | 29 (11%) | 50 (14%) | 49 (21%) | 29 (11%) |
| NYHA III | 218 (84%) | 169 (47%) | 143 (61%) | 191 (72%) |
| NYHA IV | 16 (6%) | 140 (39%) | 42 (18%) | 45 (17%) |
| Diabetes mellitus | 112 (43%) | 130 (36%) | 80 (34%) | 80 (30%) |
| Prior myocardial infarction | 18 (7%) | 68 (19%) | 28 (12%) | 16 (6%) |
| Paroxysmal atrial fibrillation | 146 (56%) | 76 (21%) | 87 (37%) | 69 (26%) |
| Persistent atrial fibrillation | 13 (5%) | 68 (19%) | 28 (12%) | 172 (65%) |
| Arterial hypertension | 182 (70%) | 223 (62%) | 192 (82%) | 209 (79%) |
| Hyperlipidemia | 151 (58%) | 212 (59%) | 124 (53%) | 148 (56%) |
| COPD* | 57 (22%) | 76 (21%) | 49 (21%) | 66 (25%) |
| PAD** | 52 (20%) | 68 (19%) | 40 (17%) | 45 (17%) |
| Prior stroke | 29 (11%) | 29 (8%) | 28 (12%) | 29 (11%) |
| Coronary Artery Disease*** | 153 (59%) | 245 (68%) | 124 (53%) | 106 (40%) |
| Previous PCI | 91 (35%) | 184 (51%) | 80 (34%) | 61 (23%) |
| Previous CABG | 62 (24%) | 79 (22%) | 40 (17%) | 37 (14%) |
| Prior valvular surgery | 13 (5%) | 7 (2%) | 7 (3%) | 13 (5%) |
| Previous mitral ring | 3 (1%) | 0 (0%) | 5 (2%) | 3 (1%) |
| Chronic Kidney Disease****** | 166 (64%) | 234 (65%) | 147 (63%) | 172 (65%) |
| Pacemaker | 39 (15%) | 43 (12%) | 30 (13%) | 50 (19%) |
| CRT****** | 75 (29%) | 108 (30%) | 9 (4%) | 21 (8%) |
| ICD****** | 125 (48%) | 202 (56%) | 16 (7%) | 24 (9%) |
| Medical therapy before valvular intervention: |  |  |  |  |
| 1. Beta blockers | 216 (83%) | 299 (83%) | 164 (70%) | 191 (72%) |
| 2. Diuretics | 242 (93%) | 338 (94%) | 208 (89%) | 244 (92%) |
| 3. ACE-inhibitors, Angiotensin receptor blockers | 153 (59%) | 198 (55%) | 138 (59%) | 138 (52%) |
| 4. Sacubitril/valsartan | 36 (14%) | 43 (12%) | 5 (2%) | 11 (4%) |
| [5. Mineralocorticoid receptor antagonist](https://www.ncbi.nlm.nih.gov/pmc/articles/PMC5801434/) | 156 (60%) | 227 (63%) | 73 (31%) | 109 (41%) |

CABG: Coronary Artery Bypass Graft; CRT: Cardiac Resynchronization Therapy; COPD: Chronic Obstructive Pulmonary Disease; ICD: Implantable Cardioverter Defibrillator; PAD: Peripheral Artery Disease; PCI: Percutaneous Coronary Intervention
* defined as previous MI/PCI/CABG; **defined as mL/min/1.73m^2^;

**Table S5. Echocardiographic characteristics of the Mitrascore cohort** (all data are reported as absolute number and percentage or mean ± standard deviation)

|  | **Cluster 1 (n=260, 23%)** | **Cluster 2 (n=360, 32%)** | **Cluster 3 (n=234, 21%)** | **Cluster4 (n=265, 24%)** |
| --- | --- | --- | --- | --- |
| Left ventricle end diastolic diameter (LVEDD,mm) | 67±11 | 69±46 | 55±8 | 55±9 |
| Left ventricle end diastolic volume (LVEDV, ml) | 214±93 | 202±69 | 117±42 | 123±51 |
| Left ventricle end diastolic volume/BSA (ml/m^2^) | 115±48 | 112±35 | 65±21 | 68±27 |
| Left ventricular ejection fraction (%) | 27±11 | 25±9 | 45±8 | 43±9 |
| Tricuspid annular plane excursion (TAPSE, mm) | 19±4 | 15±4 | 20±5 | 18±4 |
| Systolic Pulmonary pressure (sPAP mmHg) | 46±14 | 54±17 | 45±15 | 51±16 |
| Left atrial volume (LAV,ml) | 95±51 | 105±56 | 92±59 | 109±51 |
| Mitral regurgitation: |  |  |  |  |
| Moderate | 31 (12%) | 32 (9%) | 23 (10%) | 19 (7%) |
| Severe | 231 (89%) | 328 (91%) | 208 (89%) | 246 (93%) |

**Table S6. Baseline characteristics of the optimal medical therapy cohort** (all data are reported as absolute number and percentage or average ± standard deviation)

|  | **Cluster 1 (n=37, 18%)** | **Cluster 2 (n=96, 46%)** | **Cluster 3 (n=10, 5%)** | **Cluster4 (n=64, 31%)** |
| --- | --- | --- | --- | --- |
| Age (years old) | 72±13 | 69±13 | 84±6 | 82±9 |
| BMI (kg/m^2^) | 31±24 | 26±4 | 22±5 | 25±4 |
| NYHA classes |  | | | |
| NYHA I | 0 (0%) | 3 (3%) | 1 (10%) | 2 (3%) |
| NYHA II | 19 (51%) | 23 (24%) | 7 (70%) | 19 (30%) |
| NYHA III | 15 (41%) | 50 (52%) | 2 (20%) | 29 (45%) |
| NYHA IV | 3 (8%) | 20 (21%) | 0 (0%) | 14 (22%) |
| Diabetes mellitus | 10 (27%) | 37 (39%) | 3 (30%) | 28 (44%) |
| Prior myocardial infarction | 11 (30%) | 40 (42%) | 5 (50%) | 13 (20%) |
| Atrial fibrillation | 1 (3%) | 60 (63%) | 4 (40%) | 55 (86%) |
| Arterial hypertension | 18 (49%) | 56 (58%) | 8 (80%) | 45 (70%) |
| Hyperlipidemia | 18 (49%) | 43 (45%) | 6 (60%) | 31 (48%) |
| Smoking |  |  |  |  |
| Previous | 7 (19%) | 32 (33%) | 1 (11%) | 15 (23%) |
| Active | 6 (17%) | 7 (7%) | 1 (11%) | 1 (2%) |
| COPD* | 5 (14%) | 19 (20%) | 2 (20%) | 12 (19%) |
| PAD** | 6 (16%) | 16 (17%) | 0 (0%) | 11 (17%) |
| Prior stroke | 6 (16%) | 15 (16%) | 1 (10%) | 7 (11%) |
| Coronary Artery Disease*** | 16 (43%) | 50 (52%) | 5 (50%) | 22 (34%) |
| Previous PCI | 12 (32%) | 38 (40%) | 4 (40%) | 11 (17%) |
| Previous CABG | 5 (13%) | 20 (21%) | 2 (20%) | 8 (13%) |
| Prior valvular surgery | 2 (5%) | 4 (4%) | 0 (0%) | 10 (16%) |
| Chronic Kidney Disease****** | 20 (54%) | 63 (66%) | 7 (70%) | 37 (58%) |
| CRT****** | 10 (27%) | 36 (37%) | 0 (0%) | 5 (8%) |
| ICD****** | 18 (49%) | 60 (63%) | 0 (0%) | 6 (9%) |
| Medical therapy before valvular intervention: |  |  |  |  |
| 1. Beta blockers | 27 (73%) | 85 (89%) | 7 (70%) | 45 (70%) |
| 2. Diuretics |  |  |  |  |
| 3. ACE-inhibitors, Angiotensin receptor blockers | 12 (32%) | 25 (26%) | 3 (30%) | 20 (32%) |
| 4. Valsartan/sacubitril | 11 (30%) | 29 (30%) | 0 (0%) | 2 (3%) |
| [5. Mineralocorticoid receptor antagonist](https://www.ncbi.nlm.nih.gov/pmc/articles/PMC5801434/) | 21 (57%) | 70 (73%) | 3 (30%) | 38 (59%) |

CABG: Coronary Artery Bypass Graft; CRT: Cardiac Resynchronization Therapy; COPD: Chronic Obstructive Pulmonary Disease; ICD: Implantable Cardioverter Defibrillator; PAD: Peripheral Artery Disease; PCI: Percutaneous Coronary Intervention
* defined as previous MI/PCI/CABG; **defined as mL/min/1.73m^2^;

**Table S7. Echocardiographic characteristics of the optimal medical therapy cohort** (all data are reported as mean ± standard deviation)

|  | **Cluster 1 (n=37, 18%)** | **Cluster 2 (n=96, 47%)** | **Cluster 3 (n=10, 5%)** | **Cluster4 (n=64, 31%)** |
| --- | --- | --- | --- | --- |
| Left ventricle end diastolic diameter (LVEDD,mm) | 68±11 | 68±10 | 84±9 | 52±9 |
| Left ventricle end diastolic volume (LVEDV, ml) | 229±79 | 214±72 | 105±41 | 119±45 |
| Left ventricular ejection fraction (%) | 27±10 | 24±8 | 51±12 | 46±14 |
| Tricuspid annular plane excursion (TAPSE, mm) | 19±4 | 16±3 | 23±4 | 18±3 |
| Systolic Pulmonary pressure (sPAP mmHG | 42±11 | 52±13 | 45±15 | 54±13 |
| Left atrial volume (LAV, ml/m^2^) | 115±44 | 148±64 | 113±36 | 151±81 |
| Average E/e’ | 16±5 | 18±10 | 13±2 | 15±5 |
| Right ventricle diameter (mm)* | 42±4 | 47±11 | 45±8 | 48±7 |

Table S8. Pearson correlation coefficient between BMI and the other variables included in the clustering model

| Variables | Pearson correlation | P-value |
| --- | --- | --- |
| LVEDD | 0.07 | 0.13 |
| LVEDV | 0.05 | 0.34 |
| LVEF | 0.05 | 0.25 |
| Prior AMI | 0.04 | 0.34 |
| AF type | 0.03 | 0.44 |
| TAPSE | 0.02 | 0.66 |
| NYHA | - 0.01 | 0.73 |
| sPAP echo | - 0.06 | 0.14 |
| Age | - 0.08 | 0.06 |

**Table S9. Pearson correlation coefficient between TAPSE and the other variables included in the clustering model**

| **Variables** | **Pearson correlation** | **P-value** |
| --- | --- | --- |
| **LVEF** | **0.28** | **<0.05** |
| **Age** | **0.13** | **<0.05** |
| **BMI** | **0.02** | **0.67** |
| **sPAP echo** | **-0.08** | **0.07** |
| **LVEDD** | **-0.09** | **0.03** |
| **LEVDV** | **-0.10** | **0.04** |
| **NYHA** | **-0.13** | **<0.05** |
| **AF type** | **-0.14** | **<0.05** |
| **Prior AMI** | **-0.16** | **<0.005** |

**Sensitivity analysis for imputation method**

**In the present study we opted to use a simple imputation technique, specifically the median for continuous variables and the mode for categorical variables because most of the variables in our dataset have a very low percentage of missing data (over 95% complete), with the exception of two variables: TAPSE (30% missing) and BMI (33% missing), as reported in Supplementary Figure S1. For these variables, we used the median, a robust imputation technique, as it is less sensitive to outliers and does not rely on correlations, which in our case are weak as presented in the Supplementary tables S8 e S9, making advanced methods such as MICE less suitable. However, for completeness, we reperform the clustering using MICE as the imputation method. As reported in the new Supplementary Figure S5, the results confirm that even with MICE, our choice of the optimal number of clusters (4) remains unchanged, as determined by our evaluation metric. The cluster sizes obtained are also very similar:**

**Median 176, 218, 233, 195**

**MICE 175, 217, 250, 180**

**The average silhouette score obtained with the median and mode is 0.49, while with MICE it is slightly lower, with an average of 0.47. The silhouette scores for each cluster are as follows:**

**Median : 0.49, 0.46, 0.47, 0.56**

**MICE: 0.49, 0.42, 0.43, 0.53**

**This indicates that the imputation method does not significantly affect the distribution of samples across clusters. Regarding primary end point the results between the two imputation techniques are highly comparable**

**Median: [42.0%, 36.5%, 24.6%, 19.7%]**

**MICE: [38.9%, 36.9%, 27.4%, 20.8%]**

**These results show that, although the imputation techniques differ, the impact on clustering results and outcomes is minimal and that our choice of using a simple while robust technique is justified by a higher silhouette score in all formed groups.**
